# Technical Report of Successful Deployment of Tandem Visual Tracking During Live Laparoscopic Cholecystectomy Between Novice and Expert Surgeon

**DOI:** 10.7759/cureus.791

**Published:** 2016-09-20

**Authors:** Yana Puckett, Benedicto C Baronia

**Affiliations:** 1 Surgery, Texas Tech University Health Sciences Center; 2 Neurosurgery, Texas Tech University Health Sciences Center

**Keywords:** tandem visual tracking, laparoscopic cholecystectomy, surgical education, visual tracking

## Abstract

With the recent advances in eye tracking technology, it is now possible to track surgeons’ eye movements while engaged in a surgical task or when surgical residents practice their surgical skills. Several studies have compared eye movements of surgical experts and novices and developed techniques to assess surgical skill on the basis of eye movement utilizing simulators and live surgery. None have evaluated simultaneous visual tracking between an expert and a novice during live surgery. Here, we describe a successful simultaneous deployment of visual tracking of an expert and a novice during live laparoscopic cholecystectomy.

One expert surgeon and one chief surgical resident at an accredited surgical program in Lubbock, TX, USA performed a live laparoscopic cholecystectomy while simultaneously wearing the visual tracking devices. Their visual attitudes and movements were monitored via video recordings. The recordings were then analyzed for correlation between the expert and the novice. The visual attitudes and movements correlated approximately 85% between an expert surgeon and a chief surgical resident. The surgery was carried out uneventfully, and the data was abstracted with ease. We conclude that simultaneous deployment of visual tracking during live laparoscopic surgery is a possibility. More studies and subjects are needed to verify the success of our results and obtain data analysis.

## Introduction

**Figure 1:**
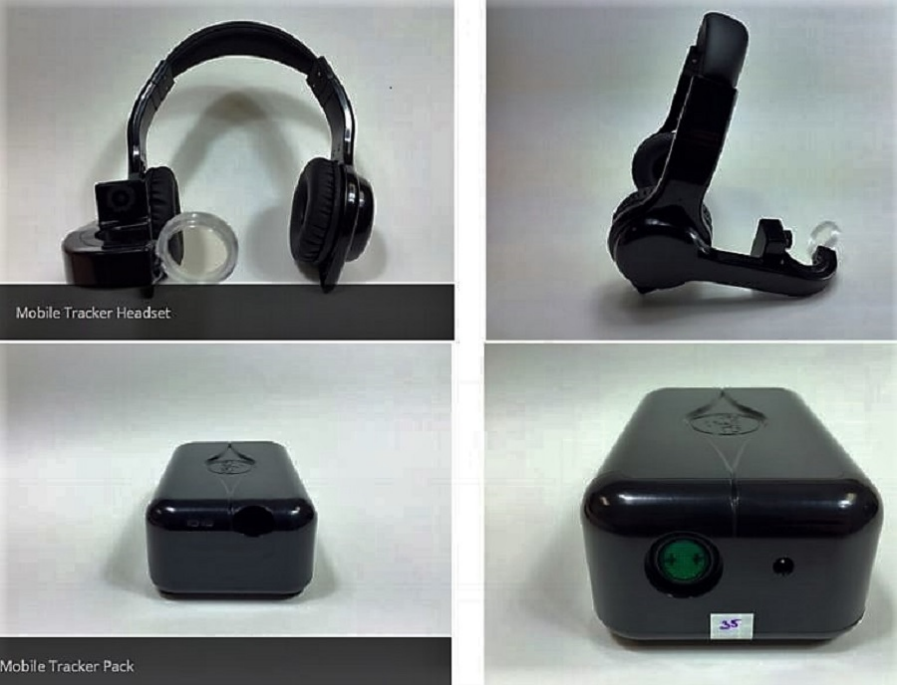
The sample unit of EyeGuide® Mobile Tracker headset and pack worn during the surgery by both the surgeons.

It is estimated that Americans undergo an average of 9.2 surgical procedures per lifetime [1]. As such, training of surgeons to obtain excellent and safe surgical skills during surgical training in residency is a public health necessity. Surgical residency is typically five years long. Throughout that time, a surgical trainee acquires skills in the operating room that they will be using for the rest of their careers. With the 80-hour-work week restrictions that were put in place for surgical residents in 2003, surgical training has been criticized for not providing as much experience as the previous generations of surgeons received before the 80-hour-work week [2-5]. A solution to improve the technical skills of the current graduating surgeons could be the use of visual tracking devices (Figure 1). Modern technology has made it possible to track visual attitudes between the expert and the novice using visual tracking devices [6-9]. The primary endpoints of visual tracking are steady eye gaze rate, fixation and saccades duration, and designated areas of interest in the subject's visual field. The secondary endpoints are maximum pupil size, change frequency in pupil size, and predictability of pupil change [10-14].

Eye tracking technology applies video analysis technology to allow for the non-intrusive measurement of an observer’s point of gaze for a range of tasks (such as visual search, reading, and economic decision making) and a range of settings (such as in driving, motor tasks, and social interactions) [8, 10, 12]. These developments have not gone unnoticed by researchers interested in skill acquisition in surgery. Previous studies have been performed utilizing eye tracking, particularly in sports training, pilot training, and post-concussion research [5-6]. In all these studies, it is evident that the experts showed more target gaze behavior whereas the novices more often demonstrated switching and tool following behavior, thereby suggesting more pro-active gaze behavior in the experts. However, no study has ever been done where the expert surgeon and the novice surgeon operate live while their eye patterns are being recorded. We believe that by studying the eye tracking attitudes of an expert surgeon, a novice surgeon can improve his or her skills post-assessment. Here, we present for the first time a technical report where a visual tracking device was deployed in tandem during laparoscopic cholecystectomy.

## Technical report

**Figure 2:**
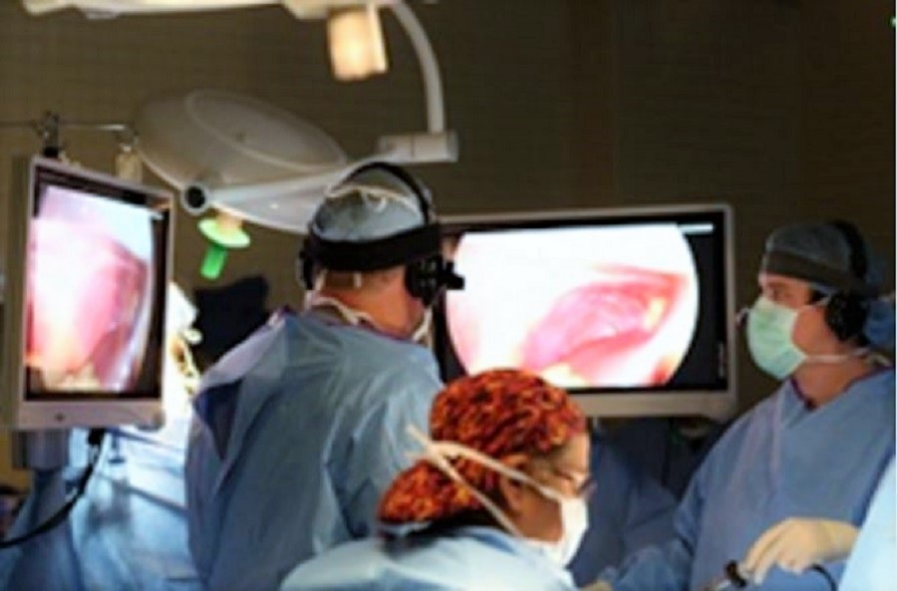
Picture obtained during surgery while the surgeon attending (expert) and the surgical resident (novice) performed a laparoscopic cholecystectomy as the EyeGuide® tracking device records the information.

Two surgeons at the University Medical Center in Lubbock, Texas, USA used paired EyeGuide® Mobile Trackers (Grinbath, Lubbock, TX) to document visually what they viewed as they carried out laparoscopic gallbladder removal (Figure 2). The headsets were applied prior to the surgical scrub. The video cameras were installed prior to the surgery. The expert surgeon was an attending board-certified physician while the novice was a chief surgical resident. The data was collected and compared for correlation. A correlation score of 85% was achieved between the surgeon expert and the surgeon novice. Further analysis is needed with more subjects to obtain the statistical significance, the average time for completion, and the patterns of novice versus expert in live surgery. The primary endpoints of visual tracking were steady eye gaze rate, fixation and saccades duration, and designated areas of interest in the subject's visual field. The secondary endpoints were maximum pupil size, change frequency in pupil size and predictability of pupil change.

The expert demonstrated higher fixation frequency, dwell time on the operative site during the application of clips and during dissection of critical view of safety, and dwelled more on the sterile field during the removal of the gallbladder out of the port site. No subjects reported problems with wearing the device or obstruction of view.

## Discussion

The rationale for the simultaneous visual tracking approach using the combined EyeGuide® Mobile Trackers centers on the 'quiet eye' (QE) concept [8-9]. Experts in activities, such as sports, which are highly dependent on effective visualization, will often have much better, concentrated focus than novices. Before carrying out a critical task, such as hitting a baseball or performing the next step in a surgical procedure, the expert will subconsciously eliminate distraction and quiet the eye to focus vision only on that which is most important to be viewed for completing the task successfully [5-7]. Novices, however, have a quicker, unfocused (saccadic) or a less quiet eye, which leads to a delayed execution, if not, also more error. The expectation of simultaneous visual tracking is that the novice surgeon’s quiet eye will evolve faster than it would through conventional training to match the expert surgeon’s quiet eye [1-7]. By studying the visual tracking patterns of the expert, the novice can gain valuable information on the areas that require more focus and attention during the operation and the areas where the focus is being wasted. Such information can be applied to the next operation for improved performance and speed. Certainly, this has been the case to date in sports where paired training was employed [2].

## Conclusions

Our case revealed that tandem deployment of visual tracking during live surgery is feasible and simple. No previous studies have demonstrated simultaneous utilization of visual tracking monitoring during live surgery. Currently, we are in the process of a prospective study utilizing expert/novice surgeons in tandem visual tracking during laparoscopic surgery.
